# Supplementary material for: Effects of Allium hookeri Extracts on Hair-Inductive and Anti-Oxidative Properties in Human Dermal Papilla Cells
Source: Plants (Basel). 2023 May 8;12(9):1919. doi: 10.3390/plants12091919 (PMC10181221; doi:10.3390/plants12091919)

Figure S1. Effects of alliin on cell viability and hair-inductive properties genes in HDPCs. **(A)** HDPCs were treated with the indicated concentrations of alliin for 24 h and 48 h, respectively. Cell viability of HDPCs was measured via WST-1 assay. (B) mRNA level of hair-inductive genes (*VCAN*, *ALP*, and *FGF7*) was assessed via qRT-PCR and then normalized against *GAPDH*.

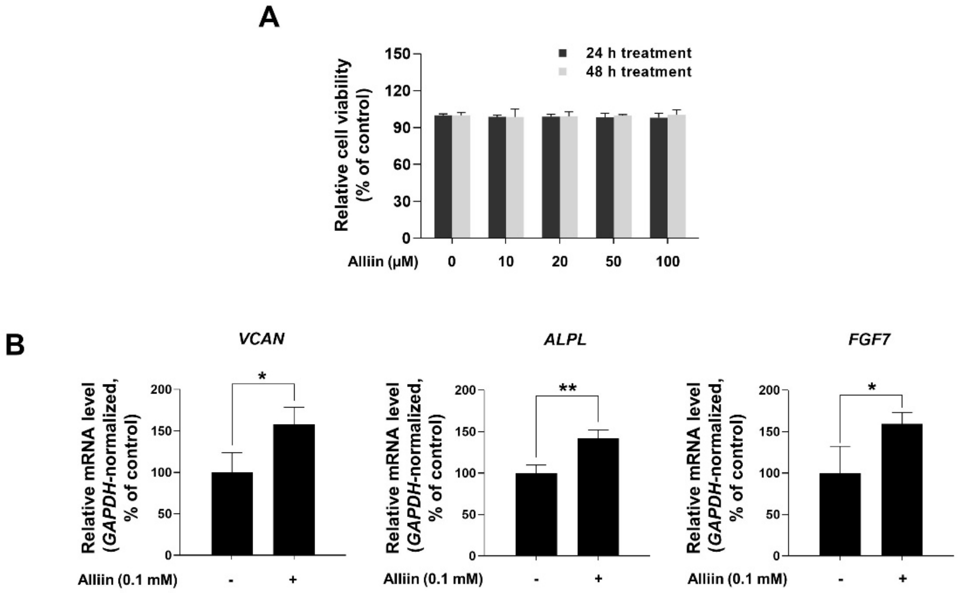

Figure S2. HDPCs were cotreated with 400 μg/mL AHE with or without 50 μM daphnetin for 24 h. The level of PKA/GSK3β signaling phosphorylation were assessed by western blotting and β-Actin served as a loading control.

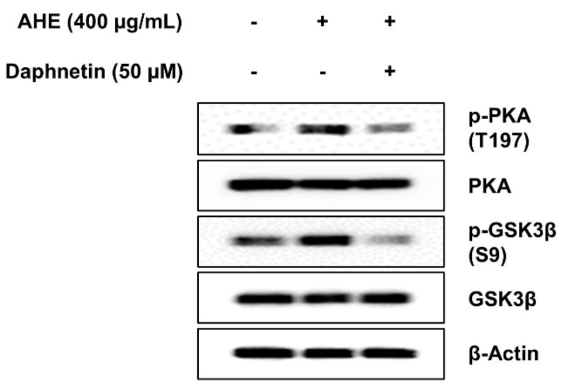

Supplement: Supplementary file 1 [file plants-12-01919-s001.zip › plants-2361345-supplementary.pdf]
